# Active sites of atomically dispersed Pt supported on Gd-doped ceria with improved low temperature performance for CO oxidation†

**DOI:** 10.1039/d3sc03988a

**Published:** 2023-10-17

**Authors:** Yuanyuan Li, Haodong Wang, Haohong Song, Ning Rui, Matthew Kottwitz, Sanjaya D. Senanayake, Ralph G. Nuzzo, Zili Wu, De-en Jiang, Anatoly I. Frenkel

**Affiliations:** a Department of Materials Science and Chemical Engineering, Stony Brook University Stony Brook NY 11794 USA liy4@ornl.gov anatoly.frenkel@stonybrook.edu; b Chemical Sciences Division, Oak Ridge National Laboratory Oak Ridge TN 37831 USA; c Interdisciplinary Materials Science, Vanderbilt University Nashville TN 37235 USA; d Chemistry Division, Brookhaven National Laboratory Upton NY 11973 USA; e Department of Chemistry, University of Illinois Urbana IL 61801 USA; f Surface and Corrosion Science, School of Engineering Sciences in Chemistry, Biotechnology and Health, KTH Royal Institute of Technology Drottning Kristinasväg 51 10044 Stockholm Sweden; g Department of Chemical and Biomolecular Engineering, Vanderbilt University Nashville TN 37235 USA

## Abstract

“Single – atom” catalysts (SACs) have been the focus of intense research, due to debates about their reactivity and challenges toward determining and designing “single – atom” (SA) sites. To address the challenge, in this work, we designed Pt SACs supported on Gd-doped ceria (Pt/CGO), which showed improved activity for CO oxidation compared to its counterpart, Pt/ceria. The enhanced activity of Pt/CGO was associated with a new Pt SA site which appeared only in the Pt/CGO catalyst under CO pretreatment at elevated temperatures. Combined X-ray and optical spectroscopies revealed that, at this site, Pt was found to be d-electron rich and bridged with Gd-induced defects *via* an oxygen vacancy. As explained by density functional theory calculations, this site opened a new path *via* a dicarbonyl intermediate for CO oxidation with a greatly reduced energy barrier. These results provide guidance for rationally improving the catalytic properties of SA sites for oxidation reactions.

## Abstract 2

The reactivity and reaction mechanism of a Pt single atom supported on ceria can be tuned by changing its local electronic and atomic structure through Gd doping.

## Graphical abstract

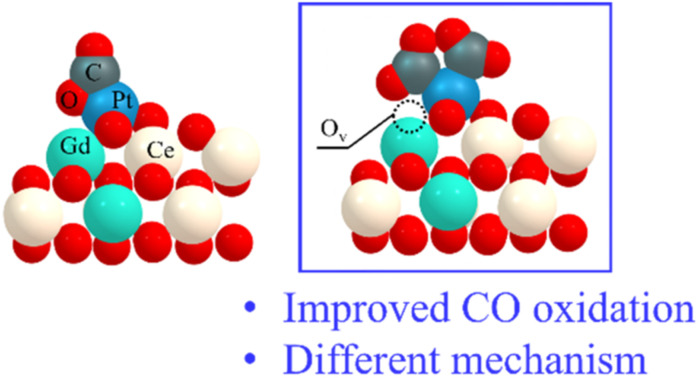

## Introduction

There is tremendous interest to increasing atom efficiency of Pt group metal (PGM)-based catalysts by exploring “single – atom” catalysts (SACs). The catalytic properties of SACs are, however, a subject of intense debate. On one hand, it was reported that SACs showed excellent activity for many reactions.^2,3^ For example, Qiao *et al.* showed that Pt_1_/FeO_*x*_ was 2–3 times more active than its nanosized counterpart in the CO oxidation reaction and preferential CO oxidation in hydrogen.^4^ On the other hand, there were results suggesting that SACs were relatively less active or even not active at all, compared to their nanoparticle counterparts.^5–11^ For example, Ding *et al.* observed that only Pt nanoparticles showed activity for CO oxidation and the water-gas shift reaction at low temperatures while Pt SAs behaved as spectators.^9^ The seemingly controversial results about their reported activities imply that SAs may exist in different geometries and therefore can be, potentially, tuned.

Indeed, there have been a large number of studies aimed at improving the thermal stability of SA systems by manipulating the interaction between singly dispersed metal atoms and the support *via* exploiting support defects.^8,12–15^ The catalytic behaviour of SAs can also be tuned by applying different pretreatment conditions.^16–22^ For example, Nie *et al.* studied Pt/ceria SACs for the CO oxidation reaction and found that the as-prepared catalyst was not active at low temperatures while after steam treatment at 750 °C, the catalyst started to show activity at 60 °C.^22^ In addition, the SA species, and their electronic/atomic structure and structure evolution can be tuned by modifying the support. For instance, we found that when supported on nanosized ceria, only Pt^2+^ SA species were detected while on bulk ceria and Gd doped bulk ceria, Pt^2+^ SA species coexisted with a small amount of Pt^4+^ SA species.^1,23^ These Pt^2+^/Pt^4+^ SA species on these ceria supports behaved differently under temperature dependent reducing conditions.^23^ On the other hand, a Pt^4+^ SA can be the main species due to the adsorption of O_2_ but only Pt^2+^ remained after heating in He.^24^ All these results, on one hand, show the complexity of SA systems (heterogeneity of sites and sensitivity to external conditions) and on the other hand, emphasize the importance of correlating structure evolutions of SA species with their catalytic behaviours for designing SA sites with the desired structures and properties.

We recently discovered that the perimeter Pt–O vacancy (*V*_O_)–Ce^3+^ sites in a Pt/ceria nanocatalyst behaved uniquely: they remained dynamically mobile under reaction conditions compared to the rest of the Pt sites in the nanoparticle.^25^ Inspired by this observation, we propose that the dynamicity of SA sites should be controlled to enhance their catalytic performance under reaction conditions. We hypothesize that dynamicity of a SA can be improved by anchoring it on a defective ceria site and enhancing the oxygen mobility in ceria. To test our hypothesis, in this work, we focused on two SACs: Pt supported on ceria (Pt/ceria) and on Gd doped ceria (Pt/CGO). The reason for the choice of the CGO support is based on the fact that Gd^3+^ introduces oxygen vacancies into the ceria support and the oxygen mobility in CGO is high.^26^ Those properties are, in fact, the main reason why CGO has displayed outstanding electromechanical properties, as demonstrated in our previous work.^27–29^

In this work, we performed comparative studies of Pt/ceria and Pt/CGO catalysts for a model reaction of CO oxidation. To determine the nature of active Pt SA sites in Pt/ceria and Pt/CGO catalysts, scanning transmission electron microscopy (STEM), operando diffuse reflectance infrared Fourier-transform spectroscopy (DRIFTS), ambient pressure X-ray photoelectron spectroscopy (AP-XPS), and operando X-ray absorption spectroscopy (XAS) were combined to monitor catalysts under temperature dependent CO conditions and the subsequent reaction conditions. The DFT calculations were performed to provide insights into the structure–property relationship of SA species and their working mechanisms.

## Results and discussion

**Fig. 1:**
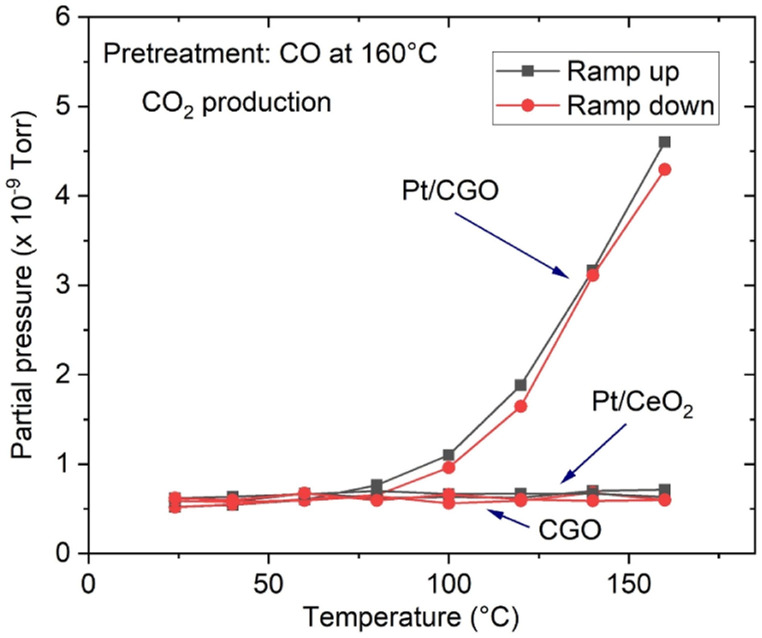
CO_2_ production over Pt/CGO and Pt/ceria. Catalysts were exposed to CO at 160 °C prior to the CO oxidation reaction. For reference, the activity result for Gd-doped ceria without Pt species was included. The as-prepared and pretreated Pt/ceria and bare CGO support showed no activity at low temperatures (≤160 °C) and the curves overlapped.

The as-prepared catalysts were first tested for the CO oxidation reaction. As shown in Fig. S1,† the as-prepared Pt/CGO SAC was not active at low temperatures (<150 °C). A similar result was also observed for the as-prepared Pt/ceria SAC (Fig. 1) by us and others.^22^ Interestingly, when Pt/CGO was exposed to CO at elevated temperatures (160 °C and 200 °C), the catalyst started to show activity at ∼60 °C in the subsequent CO oxidation reaction (Fig. S1†). It was also noticed that the higher the pretreatment temperature in CO, the higher the activity of Pt/CGO in the CO oxidation reaction (Fig. S1†). To exclude the influence of the CGO support, the CGO support without Pt was also tested and showed no activity at low temperatures (Fig. 1). After the reaction, the used Pt/CGO catalysts were collected and characterized *via* aberration corrected high-angle annular dark-field STEM (HAADF-STEM). No clustered Pt was observed in the used Pt/CGO catalysts (Fig. S2†), suggesting that the Pt species in Pt/CGO most likely remained as SAs after the reaction (also suggested by DRIFTS as detailed later). A different phenomenon was observed in Pt/ceria. As shown in Fig. 1, after heating Pt/ceria in CO at 160 °C, the catalyst was still inactive at low reaction temperatures. Increasing the pretreatment temperature to 200 °C in CO, CO conversion was observed in Pt/ceria which was, however, correlated with the formation of Pt nanoparticles (Fig. S3†). These observations suggested that Pt SA species in Pt/CGO and Pt/ceria were different and experienced different structural dynamics, resulting in different catalytic behaviours. To examine the structure evolution of the catalysts, *operando* DRIFTS and XAS were applied.

**Fig. 2:**
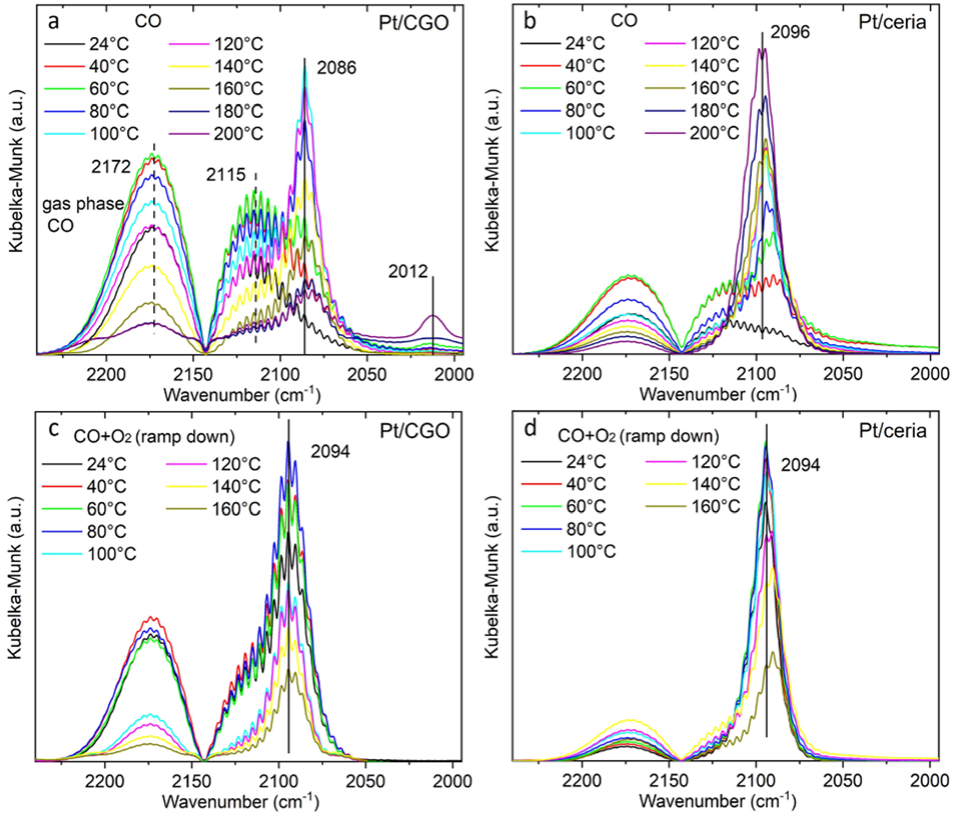
The temperature dependent DRIFTS spectra of (a) Pt/CGO and (b) Pt/ceria under CO, and those of (c) Pt/CGO and (d) Pt/ceria under CO oxidation. For (c and d), the catalysts were pretreated in CO at 160 °C prior to CO oxidation. Fig. 2(a) with a broader wavenumber range was plotted as shown in Fig. S7,† showing the absence of the bridge CO–Pt band (∼1835 cm^−1^)^1^ and indicating the atomic dispersion of Pt atoms in the pretreated Pt/CGO.

Since Pt/CGO and Pt/ceria performed differently after being pretreated in CO at different temperatures, we used DRIFTS to study both catalysts in a CO atmosphere as a function of temperature. For Pt/CGO (Fig. 2a), except for the peaks corresponding to the gas phase CO (2172 cm^−1^ and 2115 cm^−1^), the main feature observed was the peak located at about 2086 cm^−1^. This peak was assigned to linearly adsorbed CO on Pt^2+^ SA sites and it is located at a higher wavenumber (2096 cm^−1^) in the DRIFTS spectra of Pt/ceria (Fig. 2b), suggesting that the electronic features of Pt SAs in Pt/CGO were most likely modified due to Gd dopants, which caused increase of density of d electrons in Pt^2+^ SA species in Pt/CGO.^4,23,30–34^ The peak at 2086 cm^−1^ initially increased with temperature (up to 100 °C) due to the progressive reduction of Pt^4+^ to Pt^2+^.^23^ For Pt/CGO, a peak at about 2012 cm^−1^ was also observed. It was weak/invisible at low temperatures but increased steadily starting at about 160 °C (Fig. 2a). Combined with the activity results (Fig. S1†), the 2012 cm^−1^ peak could be correlated with the activity of Pt/CGO in CO oxidation: when this peak emerged, the catalyst showed activity in the subsequent CO oxidation. This peak, interestingly, cannot be found in the DRIFTS spectra of pretreated CGO (Fig. S4†) and Pt/ceria (Fig. 2b), indicating that it was associated with a unique Pt site in Pt/CGO. There are no reports, to the best of our knowledge, about the 2012 cm^−1^ peak in previous studies of Pt SA systems. One possibility is that it could be associated with small Pt or oxidized Pt clusters. However, based on previous reports, Pt metallic and oxidized clusters would show broad and asymmetric CO–Pt bands, which were not observed in this work.^35,36^ In our previous work on a Pt/ceria nanocatalyst, a band centered at ∼2016 cm^−1^ was observed, and it was assigned to CO linearly adsorbed on a perimeter Pt^0^–*V*_O_–Ce^3+^ site.^25^ We hypothesize that the 2012 cm^−1^ peak observed in Pt/CGO was associated with Pt SAs (Pt^*δ*+^: *δ* < 2) with nearby O vacancies.

As shown in Fig. 2c, the 2012 cm^−1^ peak disappeared after exposing Pt/CGO (pretreated in CO at 160 °C) to CO oxidation. Such a phenomenon was also observed for Pt/CGO being pretreated in CO at 200 °C (Fig. S5a†). In addition, when combined with the activity results (Fig. 1 and S1†), it was found that when Pt/CGO was active, there was a significant blueshift of the CO–Pt^2+^ band when the conditions were changed from CO to CO oxidation (Fig. 2a, c and 5a). The blueshift (from 2086 to 2094 cm^−1^ for the active Pt/CGO catalyst) indicated that Pt^2+^ SA sites donated electrons when the conditions changed from CO to CO oxidation.^33,37^ Such charge transfer was not observed for the Pt/CGO pretreated in CO at 120 °C (Fig. S5b†) and an inactive Pt/ceria catalyst (Fig. 1 and 2b, d). For Pt/CGO, the band at 2094 cm^−1^ shifted back to ∼2090 cm^−1^ when the reactants were replaced by He (Fig. S6†). After the reaction, the symmetric peak located close to 2090 cm^−1^ (Fig. S6†) suggested that the Pt species in the Pt/CGO catalyst remained as SAs after the reactions, in agreement with the STEM results (Fig. S2†). In principle, despite the absence of any signatures of ultra-small metal and/or oxide Pt clusters in our STEM, DRIFTS, XANES, EXAFS and XPS data, it is impossible to rule out that their minute fractions may be present. New strategies (*e.g.*, ref. 38 and 39) have recently been proposed, and may be applicable for this purpose, requiring a detailed, separate investigation.

**Fig. 3:**
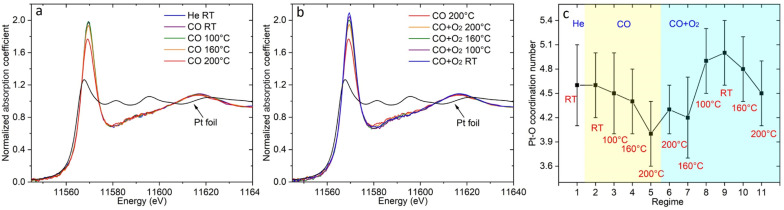
The normalized Pt L_3_ edge XANES spectra of Pt/CGO under (a) CO and (b) subsequent CO oxidation. Under CO conditions, the temperature was increased from RT to 200 °C. In CO oxidation, the temperature was decreased from 200 °C to RT. (c) The change of the Pt–O coordination number under different conditions.

To get more insights into the structure of the Pt SA species in Pt/CGO, XAS and XPS measurements were performed. The temperature dependent XAS data were first collected in CO and then under CO oxidation conditions (following similar procedures as the operando DRIFTS). Fig. 3a shows the temperature dependent change in Pt L_3_ edge XANES spectra in CO. The high intensity of the white line (2p → 5d transition) of the XANES spectra at about 11 569 eV suggested that Pt was in an oxidized state in Pt/CGO. AP-XPS spectra of Pt 4f for Pt/ceria and Pt/CGO under CO and subsequent CO oxidation conditions were also collected (Fig. S8†) with an attempt to determine the oxidation states of involved Pt species. However, due to the low weight loadings of Pt, the Pt 4f XPS data were not analysable. On the other hand, based on our previous work on Pt/ceria and Pt/CGO with higher Pt weight loadings, Pt^2+^ and a small contribution of Pt^4+^ coexisted in the as-prepared catalysts. Compared with Pt/ceria, the doping of Gd could help stabilize Pt single atoms under CO at elevated temperatures by modifying the electronic structures and geometries of Pt single-atom species and increasing the number of oxygen vacancies neighboring Pt single atoms.^23^ As evidenced by XPS results (Fig. S9–S12†), compared with Pt/ceria, in Pt/CGO, Gd^3+^ dopants caused an increase in Ce^3+^/surface defective sites and complicated O 2p hybridization.

In CO, the decrease in the onset of the white line intensity was noticed at 160 °C and became significant at 200 °C, indicating that Pt SA species on CGO gained d electrons. Such a change may correspondingly cause the redshift of the CO vibration band at 2086 cm^−1^ (Fig. 2a) in DRIFTS. However, such a shift was not observed, indicating the stability of the structure of Pt^2+^ SA sites in Pt/CGO. The observed decrease of the white line (Fig. 3a) could be then correlated with the new emerged CO vibration band at 2012 cm^−1^ (Fig. 2a). The associated lower white line intensity and the lower frequency of the CO vibration band suggested that in this new species, Pt was in a reduced *δ*+ state (*δ* < 2). After CO, the subsequent addition of O_2_ resulted in the increase of the white line as shown in Fig. 3b, suggesting that Pt single atom species donated d electrons when O_2_ was introduced into the system. Accordingly, the decrease of density of d electrons in Pt^2+^ SA species and the disappearance of Pt^*δ*+^ in Pt/CGO when the conditions changed from CO to the CO oxidation were observed from DRIFTS data (Fig. 2a and c).

The EXAFS data (Fig. S13 and S14†) were analyzed to reveal the local atomic structure of Pt SA sites in Pt/CGO. The Pt–O coordination number under different conditions is shown in Fig. 3c. For Pt/CGO, the Pt–O bond distance was about 2.00 Å (Fig. S15†), systematically longer than that in Pt/ceria (about 1.98 Å), due to nearby Gd sites and suggested that a significant fraction of Pt species was proximal to surface Gd sites.^23^ In CO (Fig. 3c and d), with the increase in the temperature, the Pt–O coordination number decreased. As discussed, since a Pt^2+^ SA site was relatively stable in CO, the changes observed in EXAFS should reflect the structural features of the new Pt^*δ*+^ site, which neighboured with less oxygen atoms compared to Pt^2+^ SA species. When O_2_ was introduced into the system, an increase in the Pt–O coordination number was observed (Fig. 3c), indicating the local structure change of the Pt^2+^ and/or Pt^*δ*+^ site. For Pt^2+^ sites, the blueshift of the CO–Pt band was also observed under CO oxidation conditions (Fig. 2a and c). Along with the increase in the Pt–O coordination number, a decrease in Ce^3+^ was observed (Fig. S11†), suggesting that the dissociated O_2_ diffused into the catalytic system.

So far, the combined results indicated that the improved catalytic activity of Pt/CGO was correlated with the appearance of Pt^*δ*+^ sites under CO conditions. To reveal the nature of this active site and to elucidate the origin of the enhanced CO oxidation activity from an atomistic view, DFT calculations were performed.

**Fig. 4:**
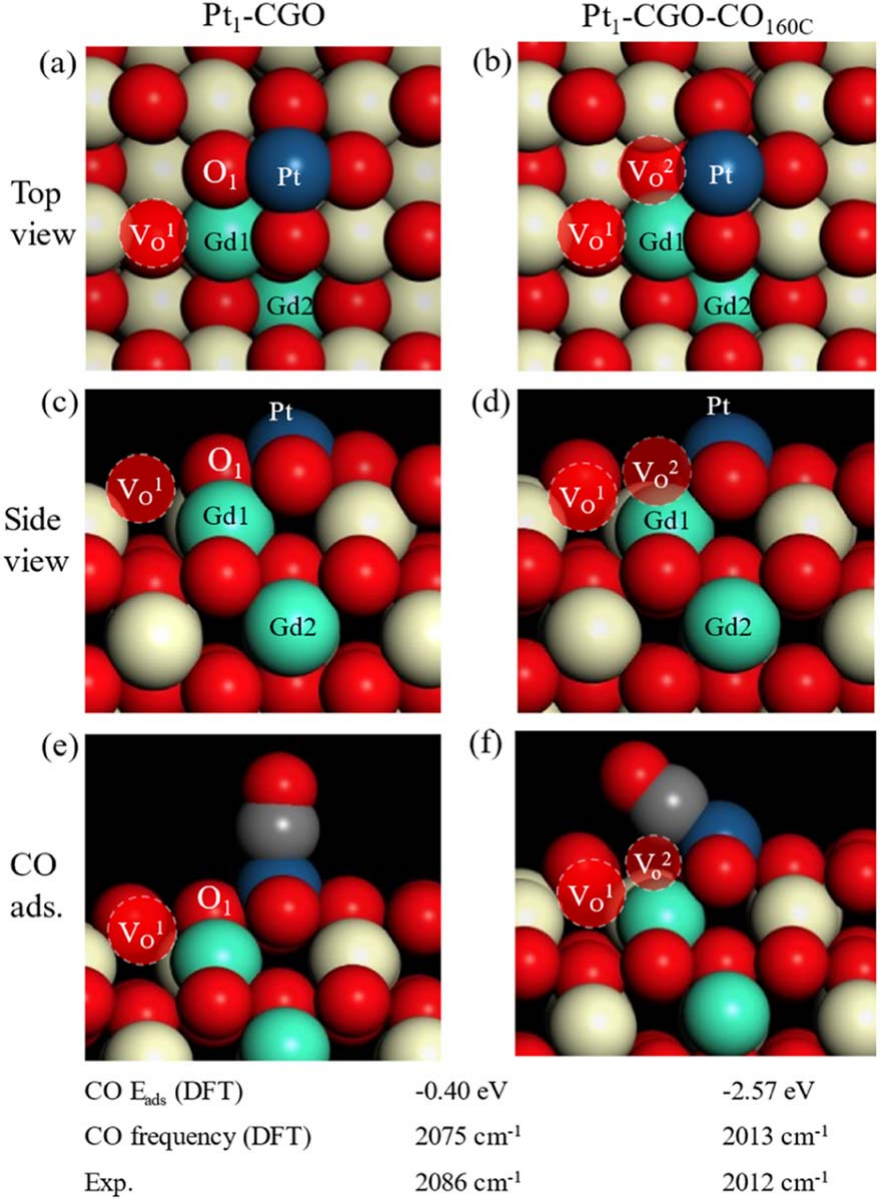
Optimized structural models for Pt_1_-CGO before (a and c) and after CO pretreatment at 160 °C (Pt_1_-CGO-CO_160C_) (b and d). CO adsorption geometry, energy, and frequencies on (e) Pt_1_-CGO and (f) CO pretreated Pt_1_-CGO. *V*_O_^1^ is introduced by Gd-doping; *V*_O_^2^ is generated from CO pretreatment.

DFT calculations were performed to generate: (1) atomistic models for as synthesized and CO-treated Pt_1_/CGO; (2) mechanisms of CO oxidation on these models. Based on the experimental information of the (100) surface of CeO_2_ being preferentially exposed,^1^ we screened CGO and then Pt_1_/CGO models by first replacing a unit of [Ce_2_O]^6+^ with [Gd_2_]^6+^ at various combinations on a slab model of CeO_2_ (100) and then placing Pt_1_ at different surface sites. After DFT search and optimization (Fig. S16 and S17†), we found the most stable models. Before CO pretreatment, the two Gd atoms were next to each other, with one in the surface Ce layer (Gd1) and the other in the subsurface Ce layer (Gd2); the intrinsic O vacancy (*V*_O_^1^) due to Gd doping was located in the surface layer next to Gd1. These results suggested that the Gd doping introduced oxygen vacancies into the surface of ceria, but these vacancies generated by Gd doping were not necessarily associated with Pt single atom sites (Fig. 4a, c and e) and thus had little effect on the activity (Fig. S1†). After CO pretreatment at 160 °C, O_1_ was removed and left a vacancy (*V*_O_^2^ in Fig. 4b and d), which bridged Pt single atom sites and *V*_O_^1^. Such a local structure around Pt single atoms was most likely responsible for the improved activity (Fig. 1) and modified CO–Pt interaction and the reaction mechanism (as discussed below). The excellent agreement between DFT and the experiment for the simulated frequencies of adsorbed CO on Pt for the two models (Fig. 4e and f) lent strong support to their appropriateness. One can see the much stronger adsorption of CO on CO-pretreated Pt_1_-CGO than on untreated Pt_1_-CGO, with a very tilted geometry due to the removal of O_1_ and the reduced state of Pt_1_.

**Fig. 5:**
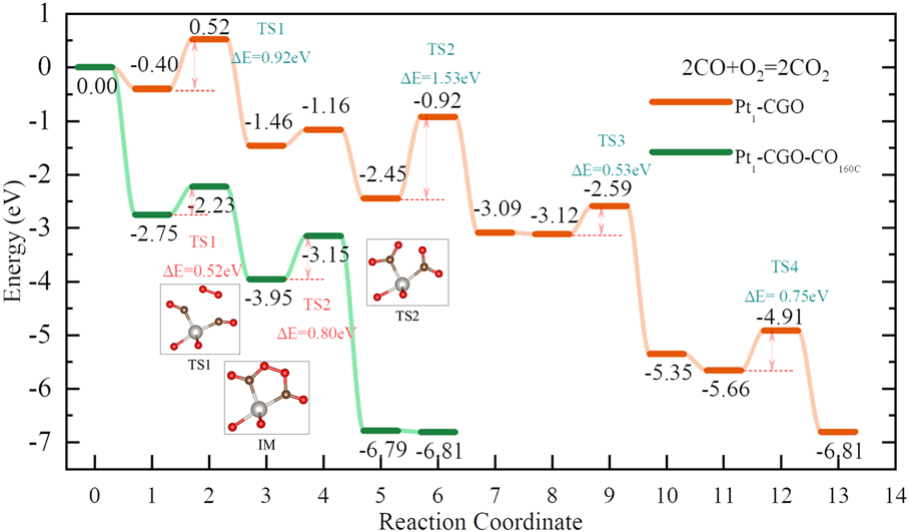
DFT-computed energy profiles of CO oxidation on Pt_1_-CGO and Pt_1_-CGO-CO_160C_.

With optimized Pt_1_-CGO and Pt_1_-CGO-CO_160C_ models, we further investigated their working mechanisms under CO oxidation. We found that CO oxidation on Pt_1_-CGO follows the conventional sequential mechanism whereby CO was oxidized one at a time and O_2_ was activated on the surface vacancies; the rate-limiting step was found to be second CO_2_ formation (TS2 orange) with a barrier of 1.53 eV (Fig. 5). In contrast, CO oxidation on Pt_1_-CGO-CO_160C_ followed a mechanism of dual–CO–adsorption, dicarbonyl-facilitated O_2_ activation, and simultaneous double CO_2_ formation.^40,41^ Both the O_2_ adsorption step to form a five-membered ring with dicarbonyl (TS1 green) and the subsequent CO_2_ formation step (TS2 green) had low activation energies (0.52 and 0.80 eV, respectively). Because the activation energy at TS1 is low (0.52 eV), we expect that the dicarbonyl intermediate will be difficult to isolate experimentally, which may explain the disappearance of the 2012 cm^−1^ band (associated with Pt^*δ*+^ sites in Pt_1_-CGO-CO_160C_) in the DRIFTS spectra under CO oxidation conditions (Fig. 2). The reason for the difficulty is that the adsorption of the second CO to form the dicarbonyl is very weak (Δ*E* = −0.18 eV) compared to physisorption strength, so it is not stable against desorption at room temperature or above in the absence of O_2_. In other words, the dicarbonyl state is a transient species which, in the presence of O_2_, quickly turns into the more stable five-membered ring intermediate (state 3 in the green profile in Fig. 4), followed by CO_2_ formation which is even more energetically favourable. Of note, the dicarbonyl intermediate has been predicted in recent computational studies of Pt single atom catalysis as well.^40,41^ Comparing Pt_1_-CGO and Pt_1_-CGO-CO_160C_ as shown in Fig. 5, one can see that CO-pretreatment of Pt_1_-CGO opened a new reaction channel of CO oxidation which had much lower activation energies *via* the dicarbonyl mechanism. The reduced Pt^*δ*+^ SA species was the key to enabling such a mechanism.

## Conclusions

In conclusion, we found that the activity of Pt/ceria can be improved by introducing Gd dopants and by CO treatment. The enhanced properties were correlated with Pt^*δ*+^ SA species, which was induced by CO at elevated temperatures. In this species, Pt had relatively weak interaction with the support, enabling the dynamic characteristics of this species. The DFT results corroborated our conjecture that Pt exhibited stronger adsorption affinity towards CGO compared to CeO_2_. Exposure of the Pt species in Pt/CGO to CO treatment generated a novel oxygen vacancy proximal to Pt SAs, engendering a new reaction mechanism *via* a dicarbonyl intermediate.

## Data availability

Sample preparation, characterization methods, DFT calculations, additional STEM images, and DRIFTS, XAS, and DFT results are provided in the ESI.†

## Author contributions

The manuscript was written through contributions of all authors. All authors have given approval to the final version of the manuscript.

## Conflicts of interest

The authors declare no conflict of interests.

## Supplementary Material

SC-014-D3SC03988A-s001
