# Supplementary material for: Active sites of atomically dispersed Pt supported on Gd-doped ceria with improved low temperature performance for CO oxidation
Source: Chem Sci. 2023 Oct 17;14(44):12582–8. doi: 10.1039/d3sc03988a (PMC10646890; doi:10.1039/d3sc03988a)
Supplement: SC-014-D3SC03988A-s001 [file SC-014-D3SC03988A-s001.pdf]

Electronic Supplementary Material (ESI) for Chemical Science.
This journal is © The Royal Society of Chemistry 2023

Electronic Supplementary Material (ESI) for Chemical Science.
This journal is © The Royal Society of Chemistry

Active Site of Atomically Dispersed Pt Supported on Gd-Doped Ceria with Improved Low Temperature Performance for CO Oxidation

Yuanyuan Li,[*a,b] Haodong Wang,[a] Haohong Song,[c] Ning Rui,[d] Matthew Kottwitz,[e] Sanjaya D. Senanayake,[d] Ralph G. Nuzzo,[e,f] Zili Wu,[b] De-en Jiang [c,g] and Anatoly I. Frenkel[*a,d]

a. Department of Materials Science and Chemical Engineering, Stony Brook University, Stony Brook, NY 11794, USA

b. Chemical Sciences Division, Oak Ridge National Laboratory, Oak Ridge, TN 37831, United States

c. Interdisciplinary Materials Science, Vanderbilt University, Nashville, TN, USA 37235

d. Chemistry Division, Brookhaven National Laboratory, Upton, NY 11973, USA

e. Department of Chemistry, University of Illinois, Urbana, IL 61801, USA

f. Surface and Corrosion Science, School of Engineering Sciences in Chemistry, Biotechnology and Health, KTH Royal Institute of Technology, Drottning Kristinasväg 51, 10044, Stockholm, Sweden

g. Department of Chemical and Biomolecular Engineering, Vanderbilt University, Nashville, TN 37235, USA.

Sample preparation

To prepare the samples, 0.50 g of either Gd-doped (20 mol%) cerium (IV) oxide nanopowder or cerium (IV) oxide nanopowder was dispersed in a solution of 2.0 g of Urea and 8.0 g water. While stirring, the desired amount of Pt precursor solution (chloroplatinic acid hydrate in water, approx. 1 wt% Pt) was added to the dispersion. All chemicals were purchased from Sigma-Aldrich. Ultrapure water (18.2 MOhm) was provided by a Millipore purification system. The mixtures were sealed in vials and stirred for 24 hours in an oil bath at 95 °C. Following the deposition process, the samples were separated and washed by 3 cycles of centrifuging/redispersing in water to remove residual ions from precursors. After drying overnight in an oven at 60 °C, the samples were crushed into powders and calcined at 500 °C (5 °C/minute heating ramp). The resultant Pt loadings were measured by Inductively Coupled Plasma (ICP) elemental analysis (Pt/Gd-CeO_2 – 0.32 wt% Pt; Pt/CeO_2 – 0.06 wt% Pt). The surface areas were measured by (Brunauer-Emmett-Teller) BET analysis of N_2 physisorption (Pt/Gd-CeO_2 – 161 m^2/g; Pt/CeO_2 – 45.2 m^2/g). The Pt surface loading was estimated to be 0.06 atoms/nm^2 for Pt/CGO and 0.04 atoms/nm^2 for Pt/ceria.

Charaterizations

Electron microscopy. Aberration corrected high-angle annular dark-field scanning transmission electron microscopy (HAADF-STEM) was conducted on an FEI Themis Z operated at 300 kV. Ex situ samples were dispersed in water and drop-casted onto lacey carbon copper grids (Ted Pella) or ultrathin carbon on lacey carbon copper grids (Graphene Supermarket). The drop-casted grids and holder were cleaned in a Tergeo EM Plasma Cleaner (remote mode, 75 W, 255 duty ratio, 20 sccm 2% H_2/bal. Ar) prior to insertion.

Operando Diffuse Reflectance Infrared Fourier-Transform Spectroscopy (DRIFTS). The spectra were collected using an iS50 FTIR spectrometer equipped with a rapid-scanning liquid nitrogen-cooled mercury cadmium telluride (MCT) detector and a Praying Mantis High Temperature Reaction Chamber (Harrick Scientific Products). In the operando setup, the outlet of the reaction chamber was connected to a mass spectrometer (Hiden QGA) which was used to detect and analyze the reactants and products. Before the measurement, the catalyst was first treated in He at 200 °C to remove water in the system and to collect the background spectra at different temperatures. Second, 1 % CO/He was introduced, and the temperature was increased stepwise from RT to a desired high temperature. Third, at the desired high temperature, 1 % CO/5 % O_2/He was introduced, and the temperature was decreased stepwise to RT. To check the reversibility of the catalyst, under the same gas condition, the temperature was increased again from RT to the desired high temperature. Fourth, at the end of the measurement, at the desired high temperature, the reaction gas mixture was replaced by pure He to examine the catalyst after the reaction. The total gas flow rate was 25 ml/min. Under each condition, the spectra were continuously collected until no further changes were observed. Each DRIFTS spectrum (256 scans) was collected with a resolution of 4 cm^{-1}. The mass spec results on the activity of catalysts were summarized in Fig. 1 and Fig. S1.

Ambient Pressure X-ray Photoelectron Spectroscopy (AP-XPS). A commercial SPECS AP-XPS instrument equipped with a PHOIBOS 150 EP MCD-9 analyzer at the Chemistry Division of Brookhaven National Laboratory (BNL) was used for XPS analysis (resolution: ~0.4 eV). A Mg K-alpha X-ray anode (1254 eV) was used for all measurements. The Ce 3*d* photoemission line with the strongest Ce^{4+} feature (916.9 eV) was used for energy calibration. The powder samples were pressed on to an aluminum plate and loaded into the AP-XPS chamber at ultra-high vacuum. For the reaction, 10 mTorr CO and subsequent 50 mTorr O_2 were introduced into the reaction chamber through a high precision leak valve and O 1 *s*, Ce 3*d,* Gd 3*d,* and Pt 4 *f* regions were collected at different temperatures.

Operando X-ray absorption spectroscopy (XAS). XAS measurements were performed at Beamline 7-BM (QAS) of National Synchrotron Light Source II, Brookhaven National Laboratory. The sample was prepared in pellets using a hydraulic press and mounted into a cylinder reactor. The Pt L_3 XAS data were collected in fluorescence mode. In the operando setup, a residual gas analyzer (Stanford SRS) was used to detect and analyze the reactants and products. In the measurement, the sample was treated in the following order: He at RT → 1 % CO/He at RT, 100 °C, 160 °C, 200 °C → 1% CO/5% O_2/He at 200 °C, 160 °C, 100 °C, RT → 1% CO/ 5% O_2/ He at 160 °C and 200 °C again. The obtained XAS data were processed and analyzed by using Demeter package. [1]

Methods of DFT calculations

The periodic density functional theory (DFT) calculations were performed using the Vienna ab initio Simulation Package (VASP).[2, 3] The Perdew-Burke-Ernzerhof (PBE)[4] functional of generalized-gradient approximation (GGA) was used for the electron exchange and correlation. The on-site Coulomb interaction was included using the DFT+U method by Dudarev, et al. in VASP using a Hubbard parameter U. Following previous existing studies using DFT+U in the literature, we use a U value of 4.5eVand 5eV [5-7] for Ce and Gd. The electron-core interaction was described using the projector-augmented wave method (PAW).[8, 9] The kinetic energy cutoff was set to 500 eV for the plane wave basis set, and the Brillouin zone was sampled using the 3×3×1 gamma point. The lattice constant is 11.48Å×11.48Å×25.53 Å, and the vacuum gap was set to 12 Å. Transition states were found using the climbing-image nudged elastic band method[10] and the dimer method[11] to achieve the final convergence and the force convergence criterion is 0.05 eV $Å^{-1}$.

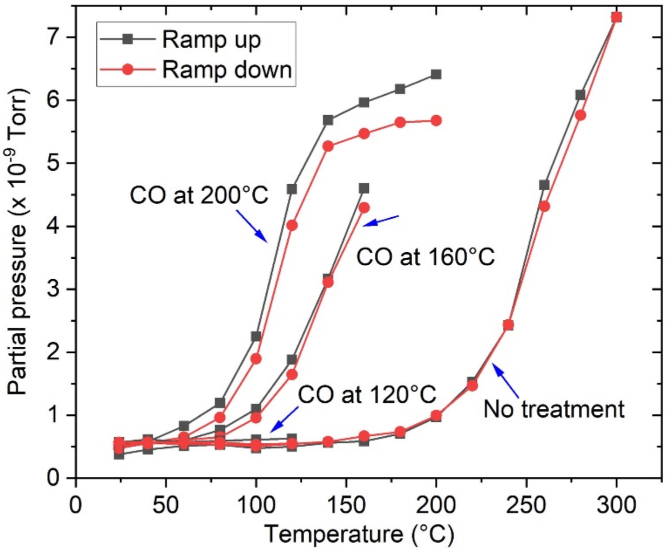

Figure S1. The temperature-dependent CO_2 production obtained via using the Pt/CGO catalyst. Before the reaction, the catalyst was heated in CO at different temperatures as indicated in the figure. Specifically, in the measurement, 1 % CO/He was introduced, and the temperature was increased stepwise from RT to a desired high temperature (120 ℃, 160 ℃, or 200 ℃). Then, at the desired high temperature, 1 % CO/5 % O_2/He was introduced, and the temperature was decreased stepwise to RT (ramp down). To check the reversibility of the catalyst, under the same gas condition, the temperature was increased again from RT to the desired high temperature (ramp up). The activity data of the as-prepared Pt/CGO was also shown here for reference.

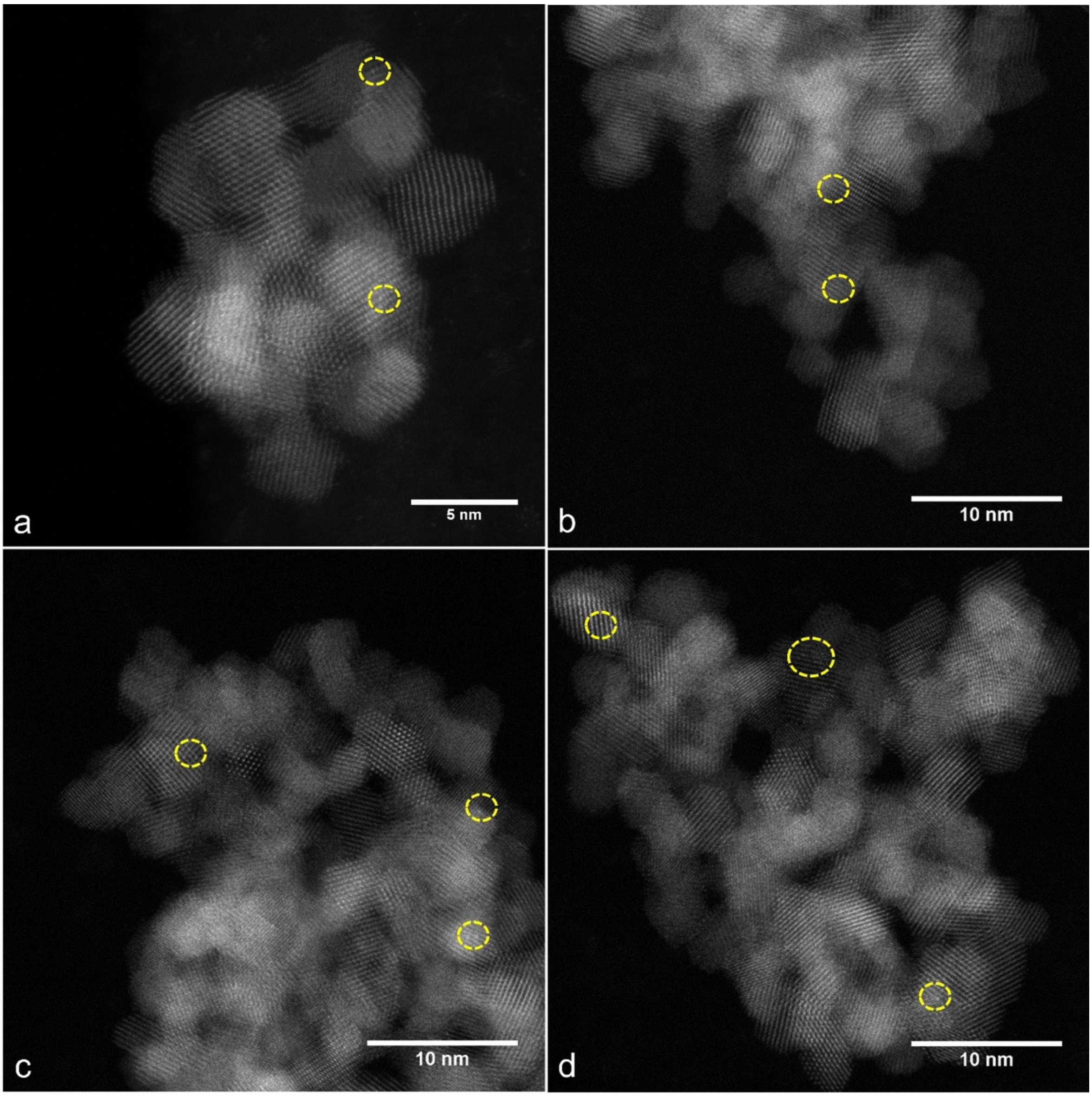

Figure S2. (a) The STEM image of the as-prepared Pt/CGO catalyst. (b-d) The STEM images of the spent Pt/CGO catalyst which was pretreated in CO at elevated temperatures (Fig. b-120 ℃, Fig. c-160 ℃, or Fig. d-200 ℃) and subsequently exposed to CO oxidation conditions.

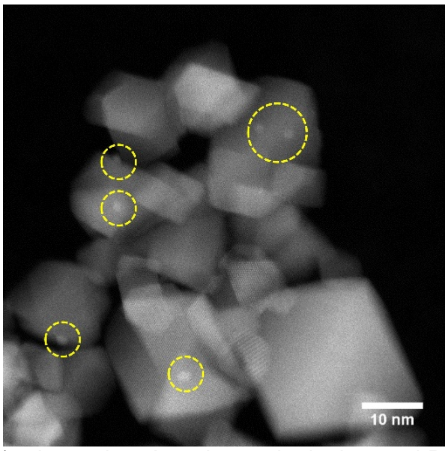

Figure S3. The STEM image of the used Pt/ceria catalyst. In order to obtain the used Pt/ceria catalyst, the as-prepared catalyst was heated in CO at 200 ℃ and experienced subsequent temperature-dependent CO oxidation reactions (the same procedure as applied to the Pt/CGO catalyst).

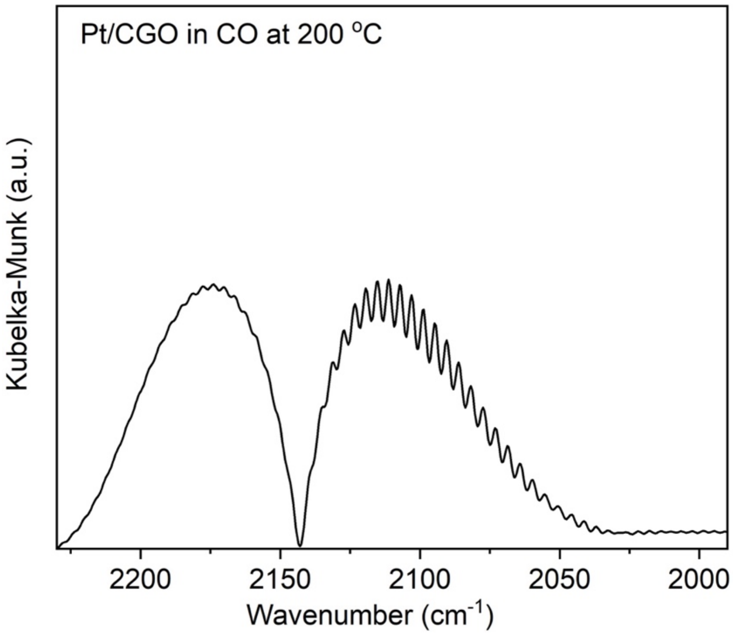

Figure S4. The DRIFTS data collected in CO at 200 °C over the bare CGO.

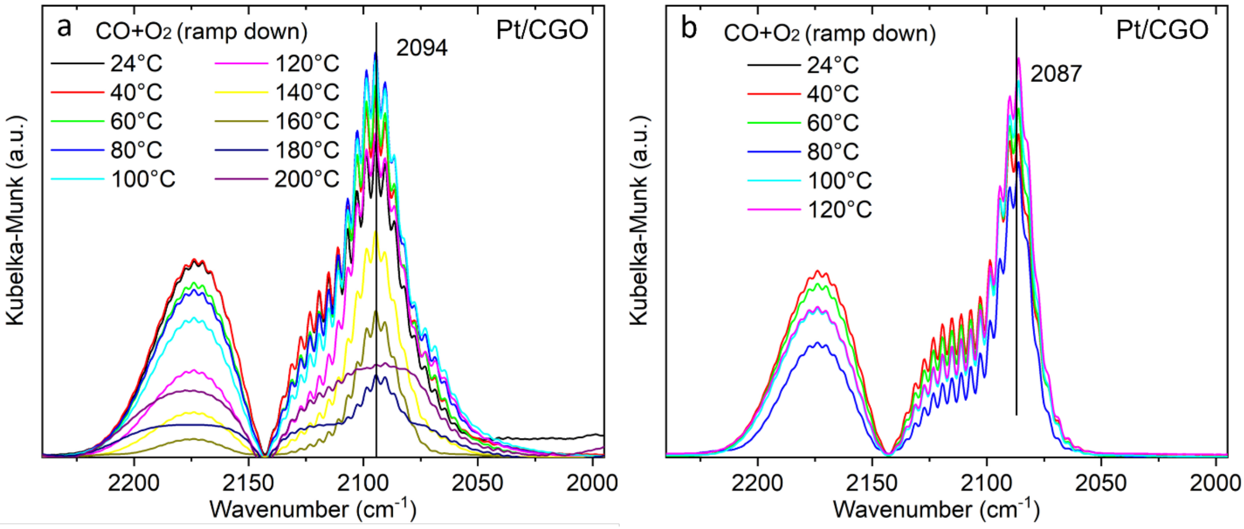

Figure S5. The DRIFTS data collected in the CO oxidation condition for the Pt/CGO catalyst pretreated in CO at (a) 200 ℃ and (b) 120 ℃. In the reaction condition, the temperature was decreased from high to RT.

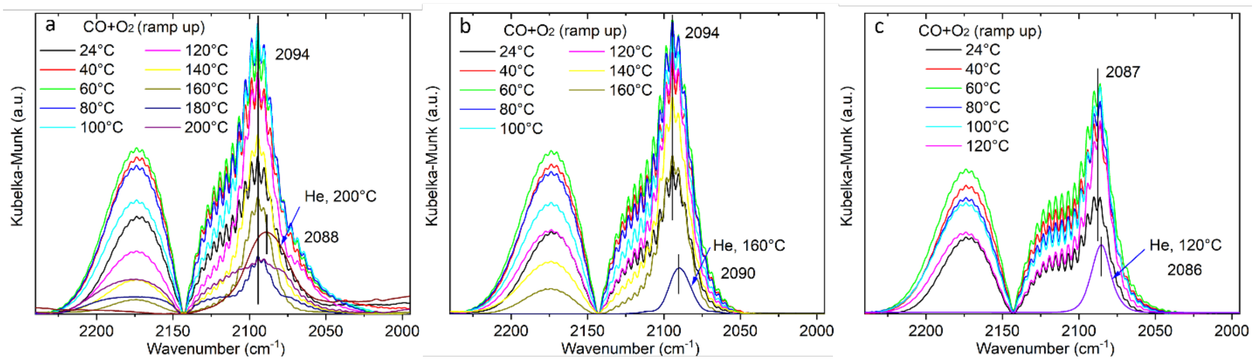

Figure S6. The DRIFTS data collected in the CO oxidation condition for the Pt/CGO catalyst pretreated in CO at (a) 200 ℃, (b) 160 ℃, and (c) 120 ℃. In the reaction, the temperature was increased from RT to high. At the end of the reaction, the reactants were flushed away by He. The corresponding data was also shown here.

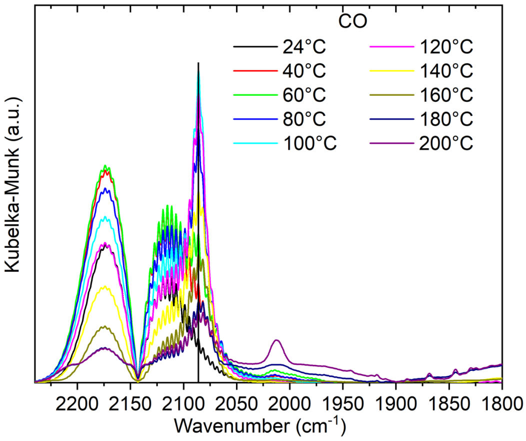

Figure S7. The temperature dependent DRIFTS spectra of Pt/CGO under CO pretreatment.

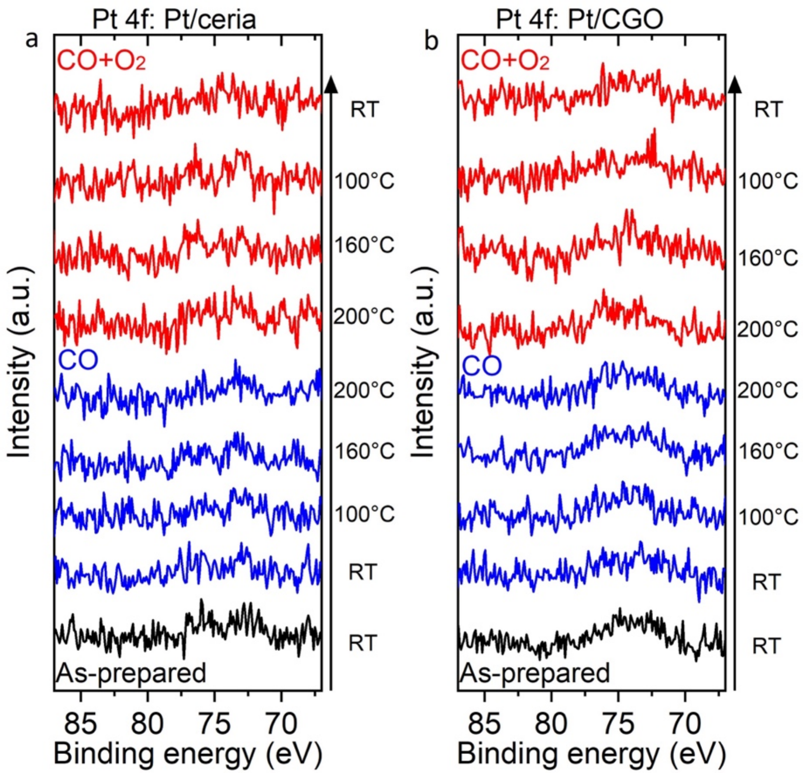

Figure S8. The Pt 4f AP-XPS of (a) Pt/ceria and (b) Pt/CGO under temperatrue dependent CO and CO oxidation conditions.

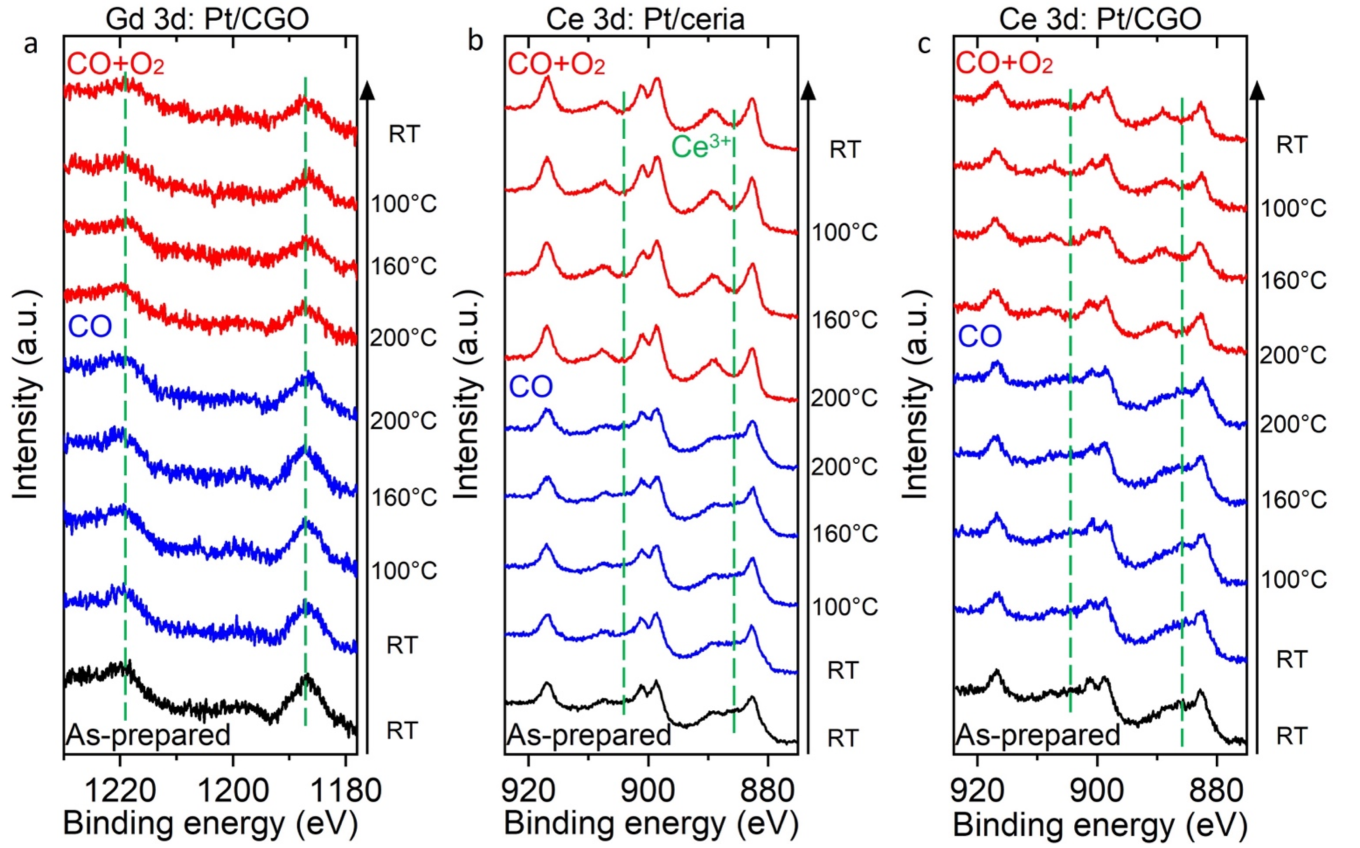

Figure S9. (a) The Gd 3d AP-XPS of Pt/CGO and the Ce 3d AP-XPS of (b) Pt/ceria and (c) Pt/CGO under temperatrue dependent CO and CO oxidation conditions. The Gd 3d AP-XPS spectra (Fig. S9a) show two peaks centered at 1187 eV and 1219 eV, corresponding to a spin-orbital splitting of $3d_{5/2}$ and $3d_{3/2}$ energy levels of Gd^{3+} respectively with the energy difference of 32 eV. These observations are in good agreement with those previously reported.[12-14]

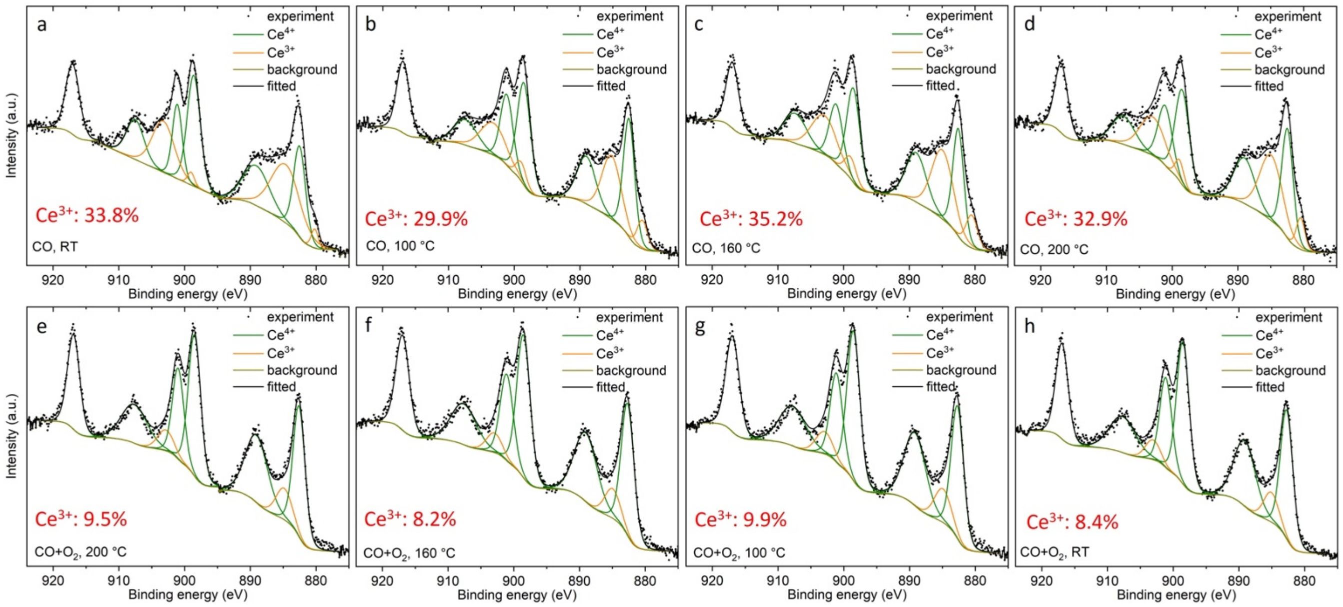

Figure S10. The experimental and fitted Ce 3d XPS for Pt/ceria under temperature dependent CO and CO oxidation conditions.

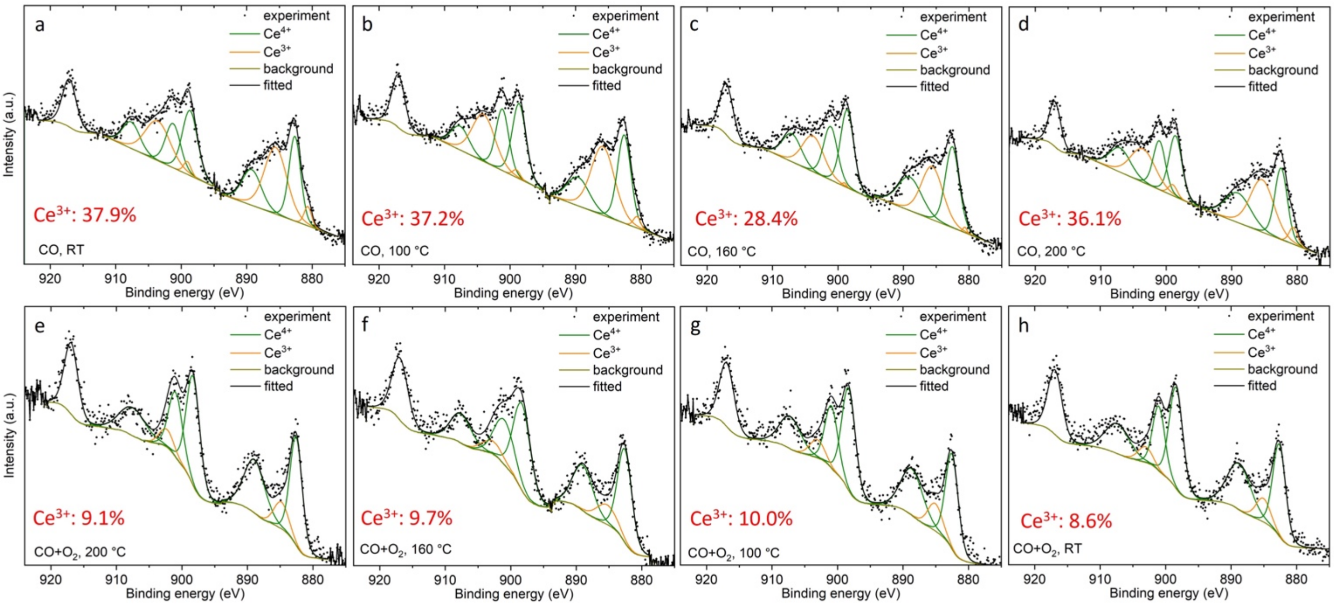

Figure S11. The experimental and fitted Ce 3d XPS for Pt/CGO under temperature dependent CO and CO oxidation conditions.

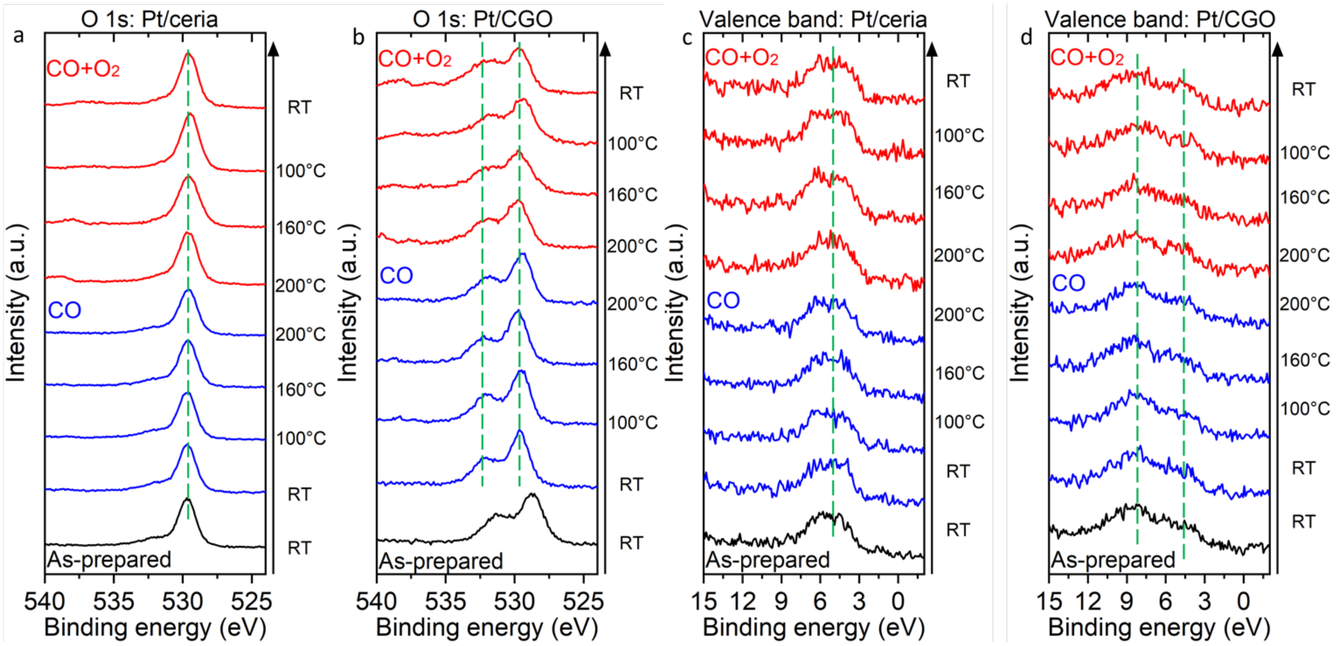

Figure S12. The O 1s AP-XPS of (a) Pt/ceria and (b) Pt/CGO and the valence band AP-XPS of (c) Pt/ceria and (d) Pt/CGO under temperatrue dependent CO and CO oxidation conditions. The O 1s and valence band XPS spectra of Pt/CGO showed different features compared with those of Pt/ceria. Specifically, the O 1s XPS spectra of Pt/ceria were dominated with a peak centered at about 530 eV that can be assigned to lattice O in oxides. In addition to this peak, a broad feature ranging from 531 eV to 533 eV was observed in the spectra of Pt/CGO and it associates with O^{2-} ions in the oxygen-deficient region and/or O from hydroxyls and/or H_2O molecules bound to Ce^{3+} species,[15-17] suggesting that the doped Gd increased the concentration of defective sites on the surface of ceria supports. Differences were also observed between the valence band XPS spectra of Pt/ceria and Pt/CGO. The valence band XPS of Pt/ceria showed a broad feature in the range of 2.0-8.0 eV, similar to that observed in the bare ceria and the broad feature is due to the hybridized O 2p states.[18, 19] For the valence band XPS of Pt/CGO, a new feature ranging from 6.0 to 12.0 eV showed up and this feature is mostly likely correlated with complicated O 2p hybridizations due to the introduction of Gd dopants.[20] Since Pt single atoms directly interact with O from the support, the modified electronic structure of supporting oxides could be the reason for the improved stability and activity of CGO supported Pt single atoms.

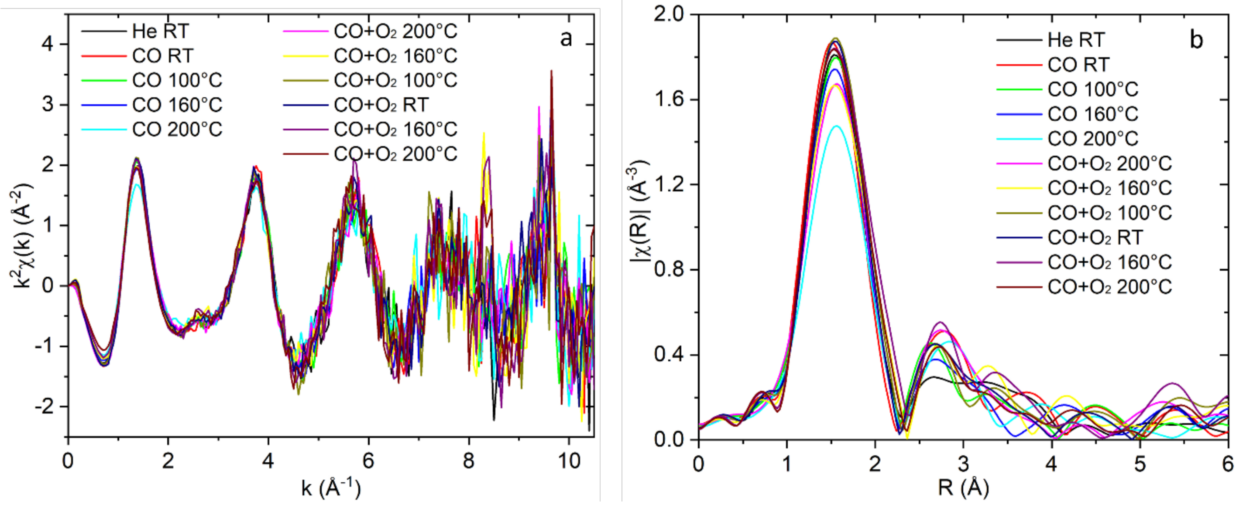

Figure S13. (a) The k space and (b) R space EXAFS data of the Pt/CGO catalyst at different conditions.

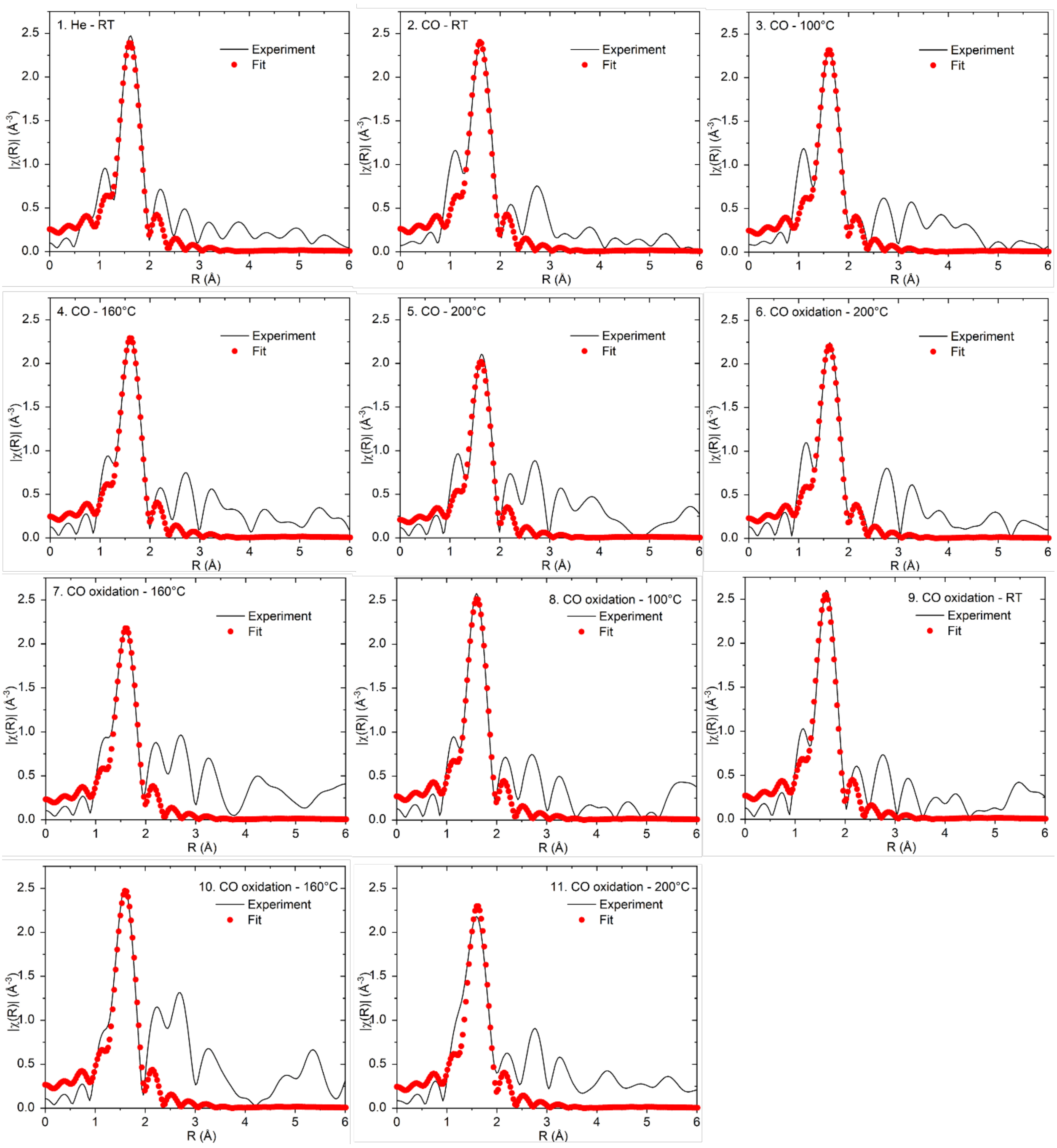

Figure S14. The comparison between the experimental EXAFS data and fit for the Pt/CGO catalyst at different conditions. As shown in Figure S13 (b), for the Pt/CGO catalyst, the pronounced peak observed at about 1.5 Å is due to Pt-O contribution. In the fitting model, the Pt-O path was then used to fit this peak. The reduction of amplitude (S_0^2: 0.84) was obtained by fitting the Pt foil. To improve the error bars, multiple data were fitted simultaneously. The shift of the threshold energy (ΔE_0) was constrained to be the same for all the data. The fitting k range is 2-11 $Å^{-1}$ and the fitting R range is 1.2-2.0 Å. The comparison between the experimental spectra and fits were plotted in Figure S8. The best fitting results on the coordination number and bond distance of Pt-O were summarized in Figure 3 (c) and Fig. S9. The obtained ΔE_0 = 12.9±0.6 eV, and σ^2 is ~ 0.000 $Å^2$.

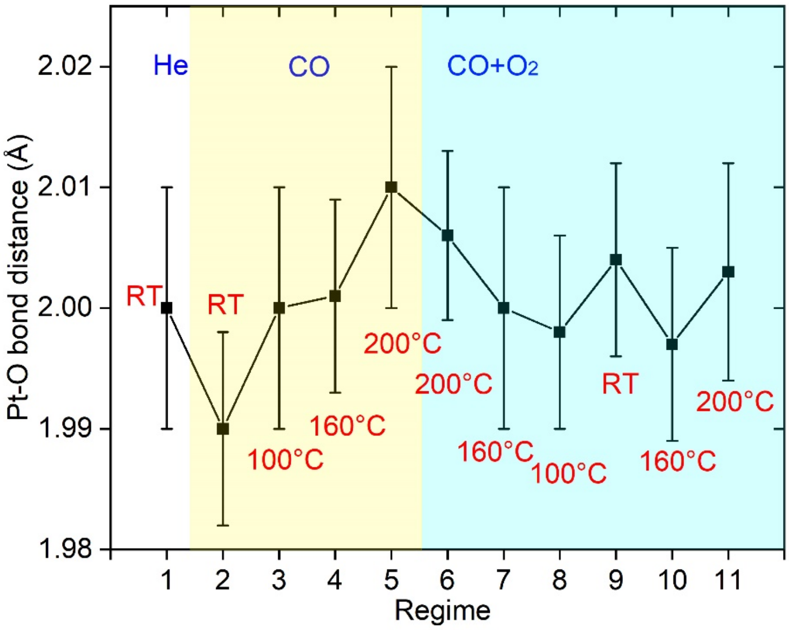

Figure S15. EXAFS-determined Pt-O bond distances of Pt/CGO catalyst under different conditions.

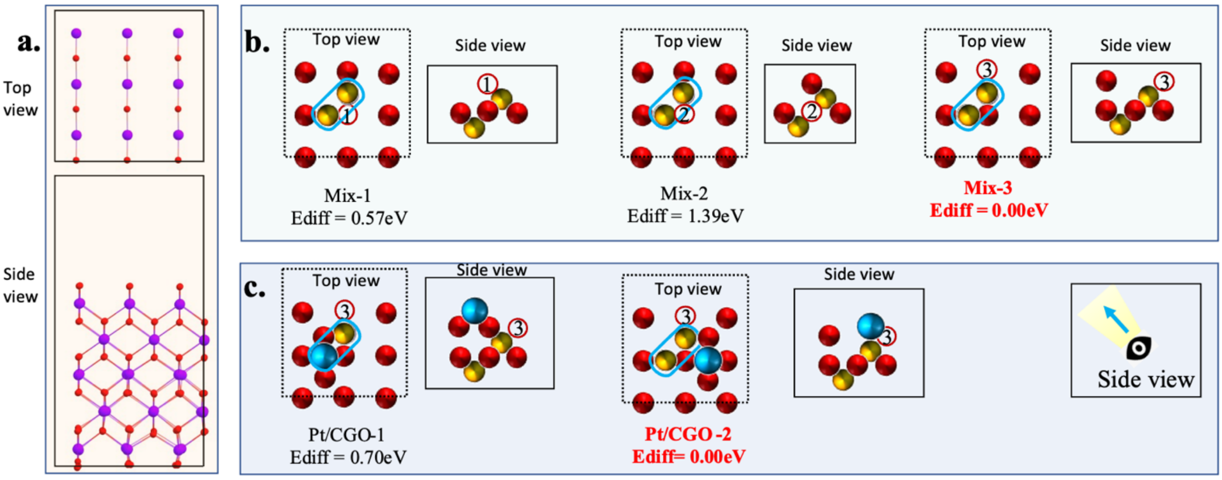

Figure S16. DFT exploration of most stable models for CGO and Pt/CGO: (a) The reconstructed (100) surface of CeO_2; (b) the best three models of CGO, created by replacing two Ce atoms and an O atom [$(Ce_2O)^{6+}$] with two Gd atoms [$(Gd_2)^{6+}$]; (c) the best two Pt/CGO models. Ce, purple; O, red; Gd, yellow; Gd, blue; O vacancy, red circle. To obtain the best CGO model, we have exhaustively explored three main scenarios: (1) exclusive Gd atom placement in the first or top layer; (2) exclusive placement in the second layer; (3) a mixed arrangement encompassing both layers. We further examined the different positioning of the O vacancy in relation to the two Gd atoms. After testing and optimizing ~30 different models, we have found the mixed configuration of one Gd in the first layer and the other in the second layer with the positioning of the O vacancy on the surface and opposite to the 2nd-layer Gd exhibits the lowest energy (the mix-3 model in Figure S16b). Notably in this model, the two Gd atoms are nearest neighbors, and the O vacancy is next to the surface Gd. From the most stable CGO model, we further explored the positioning of the Pt single atom on the surface and found the most stable one with Pt (the Pt/CGO-2 model in Figure S16c).

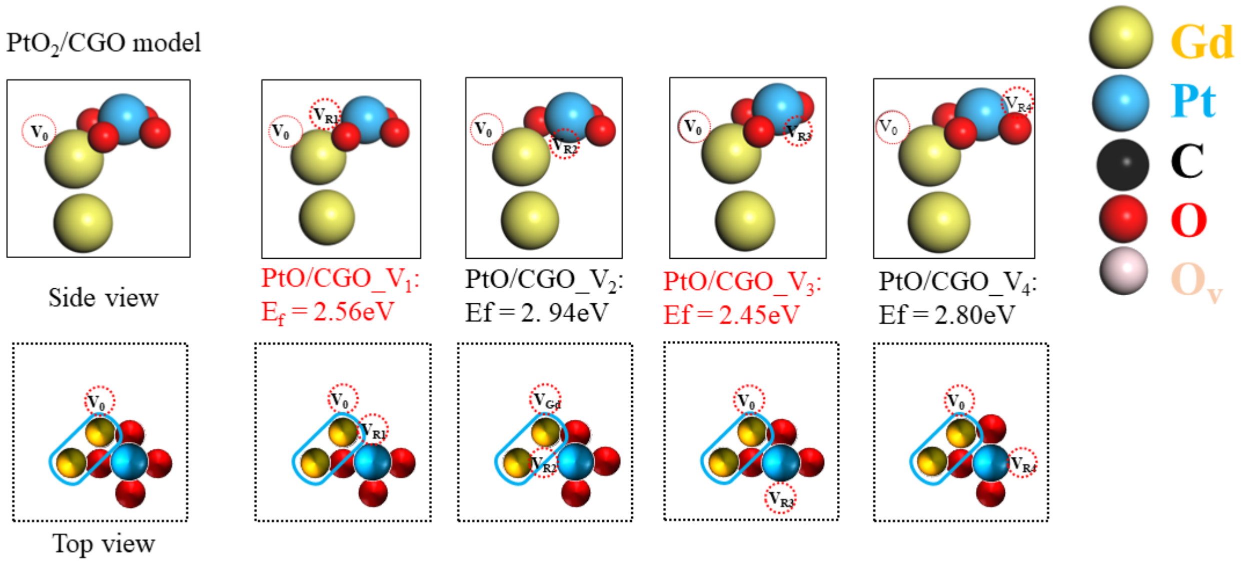

Figure S17. DFT exploration of stable models for CO-treated Pt/CGO. V_0 is the original O vacancy in CGO due to Gd doping; V_R is the O vacancy created due to the reduction of the surface by CO treatment. Based on the most stable model of Pt/CGO (the Pt/CGO-2 model in Figure S17c), we proceeded further to screen the CO-pretreated Pt/CGO model. We posit that the CO treatment reduces Pt by removing a nearby O atom, thereby giving rise to a new oxygen vacancy. So we tested the four oxygen atoms around Pt and computed the O_V formation energy at each of the four O sites and found that positions 1 and 3 (corresponding to models PtO/CGO_V1 and PtO/CGO_V3, respectively, in Figure S16) have similar stability and are more stable. We next examined CO adsorption and found that the CO frequency on PtO/CGO_V_1 at 2013 cm^{-1} agrees much better with the experiment than that on PtO/CGO_V_3 (2053 cm^{-1}) for V_{R3}. So we chose the thereby leading us to identify the oxygen vacancy at V_{R1} as a prominent and primary active site. Consequently, we selected the PtO/CGO_V_1 structure as the model for CO-treated Pt/CGO.

References

1. B. Ravel and M. Newville, ATHENA, ARTEMIS, HEPHAESTUS: data analysis for X-ray absorption spectroscopy using IFEFFIT, *Journal of Synchrotron Radiation*, 2005, **12**, 537-541.
2. G. Kresse and J. Furthmüller, Efficiency of ab-initio total energy calculations for metals and semiconductors using a plane-wave basis set, *Computational Materials Science*, 1996, **6**, 15-50.
3. G. Kresse and J. Furthmüller, Efficient iterative schemes for ab initio total-energy calculations using a plane-wave basis set, *Physical Review B*, 1996, **54**, 11169-11186.
4. J. P. Perdew, K. Burke and M. Ernzerhof, Generalized Gradient Approximation Made Simple, *Physical Review Letters*, 1996, **77**, 3865-3868.
5. S. Fabris, S. de Gironcoli, S. Baroni, G. Vicario and G. Balducci, Reply to "Comment on 'Taming multiple valency with density functionals: A case study of defective ceria' ", *Physical Review B*, 2005, **72**, 237101.
6. X. Han, N. Amrane, Z. Zhang and M. Benkraouda, Interplay between Gd and oxygen vacancy on the electronic properties and defect chemistry of Gd-doped CeO2: A DFT + U study, *Chemical Physics*, 2020, **534**, 110741.

7. T. Wu, T. Vegge and H. A. Hansen, Enhanced activity for electrocatalytic H2 production through cooperative Pr and Bi co-doping of CeO2 in solid oxide electrolysis cells, *Journal of Catalysis*, 2021, **402**, 310-314.
8. G. Kresse and D. Joubert, From ultrasoft pseudopotentials to the projector augmented-wave method, *Physical Review B*, 1999, **59**, 1758-1775.
9. P. E. Blöchl, Projector augmented-wave method, *Physical Review B*, 1994, **50**, 17953-17979.
10. G. Henkelman, B. P. Uberuaga and H. Jónsson, A climbing image nudged elastic band method for finding saddle points and minimum energy paths, *The Journal of Chemical Physics*, 2000, **113**, 9901-9904.
11. G. Henkelman and H. Jónsson, A dimer method for finding saddle points on high dimensional potential surfaces using only first derivatives, *The Journal of Chemical Physics*, 1999, **111**, 7010-7022.
12. S. N. Madhuri, K. S. Hemalatha and K. Rukmani, Preparation and investigation of suitability of gadolinium oxide nanoparticle doped polyvinyl alcohol films for optoelectronic applications, *Journal of Materials Science: Materials in Electronics*, 2019, **30**, 9051-9063.
13. S. Majeed and S. A. Shivashankar, Rapid, microwave-assisted synthesis of Gd2O3 and Eu:Gd2O3 nanocrystals: characterization, magnetic, optical and biological studies, *Journal of Materials Chemistry B*, 2014, **2**, 5585-5593.
14. N. Luo, X. Tian, C. Yang, J. Xiao, W. Hu, D. Chen and L. Li, Ligand-free gadolinium oxide for in vivo T1-weighted magnetic resonance imaging, *Physical Chemistry Chemical Physics*, 2013, **15**, 12235-12240.
15. C. Barth, C. Laffon, R. Olbrich, A. Ranguis, P. Parent and M. Reichling, A perfectly stoichiometric and flat CeO2(111) surface on a bulk-like ceria film, *Sci Rep*, 2016, **6**, 21165.
16. P. M. Shah, J. W. H. Burnett, D. J. Morgan, T. E. Davies and S. H. Taylor, Ceria–Zirconia Mixed Metal Oxides Prepared via Mechanochemical Grinding of Carbonates for the Total Oxidation of Propane and Naphthalene, *Catalysts*, 2019, **9**, 475.
17. S. Soni, V. S. Vats, S. Kumar, B. Dalela, M. Mishra, R. S. Meena, G. Gupta, P. A. Alvi and S. Dalela, Structural, optical and magnetic properties of Fe-doped CeO2 samples probed using X-ray photoelectron spectroscopy, *Journal of Materials Science: Materials in Electronics*, 2018, **29**, 10141-10153.
18. M. Kottwitz, Y. Li, R. M. Palomino, Z. Liu, G. Wang, Q. Wu, J. Huang, J. Timoshenko, S. D. Senanayake, M. Balasubramanian, D. Lu, R. G. Nuzzo and A. I. Frenkel, Local Structure and Electronic State of Atomically Dispersed Pt Supported on Nanosized CeO2, *ACS Catalysis*, 2019, **9**, 8738-8748.
19. C. Balaji Gopal, M. García-Melchor, S. C. Lee, Y. Shi, A. Shavorskiy, M. Monti, Z. Guan, R. Sinclair, H. Bluhm, A. Vojvodic and W. C. Chueh, Equilibrium oxygen storage capacity of ultrathin CeO2-δ depends non-monotonically on large biaxial strain, *Nature Communications*, 2017, **8**, 15360.
20. D. A. Zatsepin, D. W. Boukhvalov, A. F. Zatsepin, Y. A. Kuznetsova, M. A. Mashkovtsev, V. N. Rychkov, V. Y. Shur, A. A. Esin and E. Z. Kurmaev, Electronic structure, charge transfer, and intrinsic luminescence of gadolinium oxide nanoparticles: Experiment and theory, *Applied Surface Science*, 2018, **436**, 697-707.
